# Bilateral subthalamic deep brain stimulation initial impact on nonmotor and motor symptoms in Parkinson's disease: An open prospective single institution study

**DOI:** 10.1097/MD.0000000000009750

**Published:** 2018-02-02

**Authors:** Sandra Kurcova, Jan Bardon, Miroslav Vastik, Marketa Vecerkova, Monika Frolova, Lenka Hvizdosova, Martin Nevrly, Katerina Mensikova, Pavel Otruba, David Krahulik, Egon Kurca, Stefan Sivak, Jana Zapletalova, Petr Kanovsky

**Affiliations:** a Department of Neurology; b Department of Neurosurgery, University Hospital and Faculty of Medicine and Dentistry, Palacky University Olomouc, Czech Republic; c Clinic of Neurology, Jessenius Faculty of Medicine in Martin, Comenius University in Bratislava and University Hospital in Martin, Slovak Republic; d Department of Biostatistics, Faculty of Medicine and Dentistry, Palacky University Olomouc, Czech Republic.

**Keywords:** deep brain stimulation, nonmotor symptoms, subthalamic nucleus

## Abstract

Numerous studies document significant improvement in motor symptoms in patients with Parkinson's disease (PD) after deep brain stimulation of the subthalamic nucleus (STN-DBS). However, little is known about the initial effects of STN-DBS on nonmotor domains.

Our objective was to elucidate the initial effects of STN-DBS on non-motor and motor symptoms in PD patients in a 4-month follow-up.

This open prospective study followed 24 patients with PD who underwent STN-DBS. The patients were examined using dedicated rating scales preoperatively and at 1 and 4 months following STN-DBS to determine initial changes in motor and nonmotor symptoms. Patients at month 1 after STN-DBS had significantly reduced the Parkinson's disease Questionnaire scores (*P = *.018) and *Scales for Outcomes* in Parkinson's disease – Autonomic scores (*P = *.002); these scores had increased at Month 4 after DBS-STN. Nonmotor Symptoms Scale for Parkinson's Disease had improved significantly at Month 1 (*P < *.001); at Month 4, it remained significantly lower than before stimulation (*P = *.036). There was no significant difference in The Parkinson's Disease Sleep Scaleat Month 1 and significant improvement at Month 4 (*P = *.026). There were no significant changes in The Female Sexual Function Index or International Index of Erectile Function. Movement Disorder Society Unified Parkinson's Disease Rating Scale, Part III scores show significant improvements at Month 1 (*P < *.001) and at Month 4 (*P < *.001).

STN-DBS in patients with advanced PD clearly improves not only motor symptoms, but also several domains of nonmotor functions, namely sleep, autonomic functions and quality of life quickly following the start of stimulation.

## 1 Introduction

Deep brain stimulation of the subthalamic nucleus (STN-DBS) has been well established over the past 20 years for the symptomatic treatment of motor complications in advanced Parkinson's disease (PD). It provides more constant and predictable benefits than pharmacological therapy. STN-DBS lessens motor fluctuations, dyskinesias, bradykinesia, akinesia, and tremor; it also reduces dopaminergic drug requirement.^[1]^ Numerous studies have documented significant improvements in motor symptoms and quality of life of PD patients after STN-DBS.^[2]^

Nonmotor symptoms (NMS) are now recognised as an integral part of the PD clinical picture, both at the early stages and throughout the whole course of the disease, and even at the very onset of the disease, before any of the classical motor symptoms develop.^[3,4]^ Recent records indicate that NMS occur in up to 100% of PD patients, influencing the degree of disability and quality of life much more than motor symptoms.^[5]^ Evidence about the effects of DBS on nonmotor symptoms in PD is still sparse and under debate.^[6–8]^ The aim of our open, prospective, single institution study was to assess the initial impact of DBS on motor and nonmotor symptoms of advanced PD. The data were collected during four-month follow-up in 24 patients with advanced, fluctuating PD who underwent STN-DBS surgery. For the assessment, validated nonmotor and motor outcome scales were used, in contrast to previous study of Wolz et al,^[9]^ who assessed the immediate effect of STN-DBS on isolated nonmotor symptoms.

## 2 Methods

### 2.1 Subjects

24PD patients (4 females/20 males) treated for advanced PD by the bilateral STN-DBS were examined and followed-up; their demographic data are in Table 1. All patients consented to undergo clinical assessments prior to surgery and stimulation, and at regular intervals afterwards. Neuropsychological and neuropsychiatric assessments of patients were performed to exclude significant psychiatric disorder and dementia.

**Table 1:**
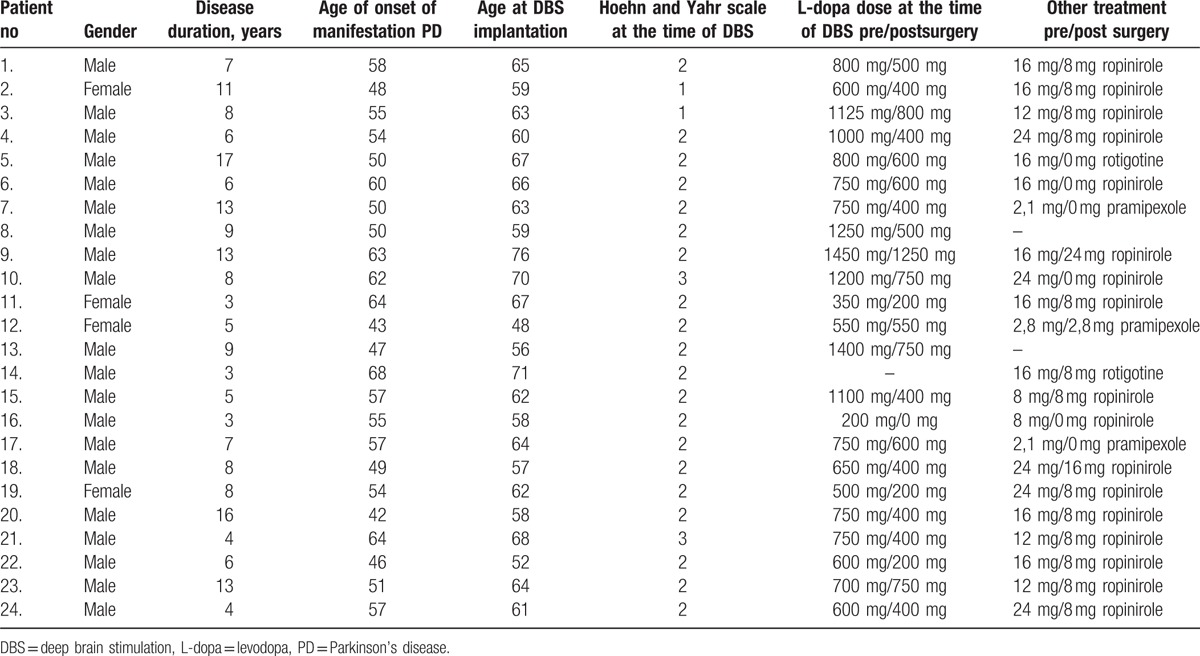
Descriptive statistics for demographic and disease-specific variables.

### 2.2 Ethical approval

The study was approved by the local ethics committee and was conducted according to the Declaration of Helsinki principles.

### 2.3 Clinical assessment

We evaluated motor symptoms and NMS preoperatively and postoperatively in PD patients utilizing the following scales:

- Movement Disorder Society Unified Parkinson's Disease Rating Scale, (MDS-UPDRS), part III: Motor Examination^[10]^
  UPDRS was originally developed in the 1980s^[11]^ and is the most widely used scale to assess impairment and disability in PD patients. MDS-UPRDS is the Movement Disorder Society's revision of UPDRS.^[10]^ We used the motor section of the UPDRS (Part III), which contains 33 scores based on 18 items, some with several scores for different body regions.
- Nonmotor Symptoms Scale for Parkinson's Disease (NMSS)^[12]^
  NMSS is a clinician-administered scale with weighted scores that tests for the frequency and severity of nonmotor symptoms (NMS) over the previous month. Severity is rated on a scale from 0 to 3 points and symptom frequency from 1 point (<once a week) to 4 points (daily or all the time). NMS are assessed in 9 domains with 30 questions. The maximum possible NMSS total score is 360; the minimum possible score is 0.
- The Parkinson's Disease Questionnaire (PDQ-39)^[13]^
  The PDQ-39 is a self-administered measure of subjective health status. It is composed of 39 items grouped in 8 subscales including mobility, activities of daily living, emotional well-being, stigma, social support, cognition, communication, and bodily discomfort.^[13]^ The time frame is “over the last month“ and responses are scored from 0 (never) to 4 (always). Subscales scores range from 0 to 100 and are obtained by transforming the total sum of the items into percentage points based on the maximum possible subscale score.^[14]^
- *Scales for Outcomes* in Parkinson's disease—Autonomic (SCOPA-Aut) Questionnaire^[15]^
  SCOPA-AUT is a self-administered questionnaire to assess dysautonomia.^[15]^ It consists of 25 items, including 3 cardiovascular, 7 gastrointestinal, 6 urinary, 4 thermoregulatory, 1 pupillomotor, and 2 sexual items, with a frequency from 0 (never) to 3 (often).
- The Parkinson's Disease Sleep Scale (PDSS)^[16]^
  The PDSS is a self-rated scale designed to measure nocturnal problems, sleep disturbance, and excessive daytime sleepiness in PD over the previous week.^[16]^ Patients evaluate 15 aspects of nocturnal and daytime sleep on a linear scale from 0 (bad) to 10 (good). The maximum possible score is 150.
- International Index of Erectile Function (IIEF)^[17]^
  The IIEF is a reliable, self-administered measure of erectile function composed of 15 items.
- The Female Sexual Function Index (FSFI)^[18]^
  The FSFI, a 19-item questionnaire, is a brief, multidimensional scale for assessing sexual function in women.^[18]^
- Jay Modified Minnesota Impulsive Disorders Interview (mMIDI)^[19]^
  The mMIDI is a self-administered questionnaire designed to screen for impulsive disorders. It is composed of 5 parts that focus on compulsive buying, compulsive gambling, compulsive sexual behavior, compulsive eating, and punding behavior.
  The data were collected preoperatively, 1 month after neurosurgery intervention, just before the adjustment of initial stimulation parameters (Month 1—M1) and 3 months after the stimulation parameters were adjusted (Month 4—M4). We used native language versions of the listed scales which were validated in previous clinical trials.

### 2.4 Statistical methods

For data analyses we used statistical software IBM SPSS Statistics version 22. The Wilcoxon rank test was used to assess the effect of DBS in modifying quantitative parameters at Month 1 and 4. The positive and negative changes in mMIDI scores were analyse dusing McNemar's test. The significance level was set at 0.05 for all tests. There was used the Bonferroni correction for multiple comparisons. Normality of data was assessed using the Shapiro–Wilk test.

## 3 Results

Twenty-four patients (4 women/ 20 men) participated in this pilot study. The demographic and disease-specific details of participants are shown in Table 1. The changes in the dosage of levodopa and dopamine agonists before STN-DBS and 4 months following the implantation are also shown there; a substantial reduction in daily dopaminergic drug requirements was present in the majority of cases. Table 2 shows the changes in the values of MDS-UPDRS III and nonmotor scales one (M1) and 4 months (M4) postoperatively (mean, median, standard deviation, minimum range, and maximum range) and also significance values based on paired Wilcoxon test or McNemar's test for mMIDI.

**Table 2:**
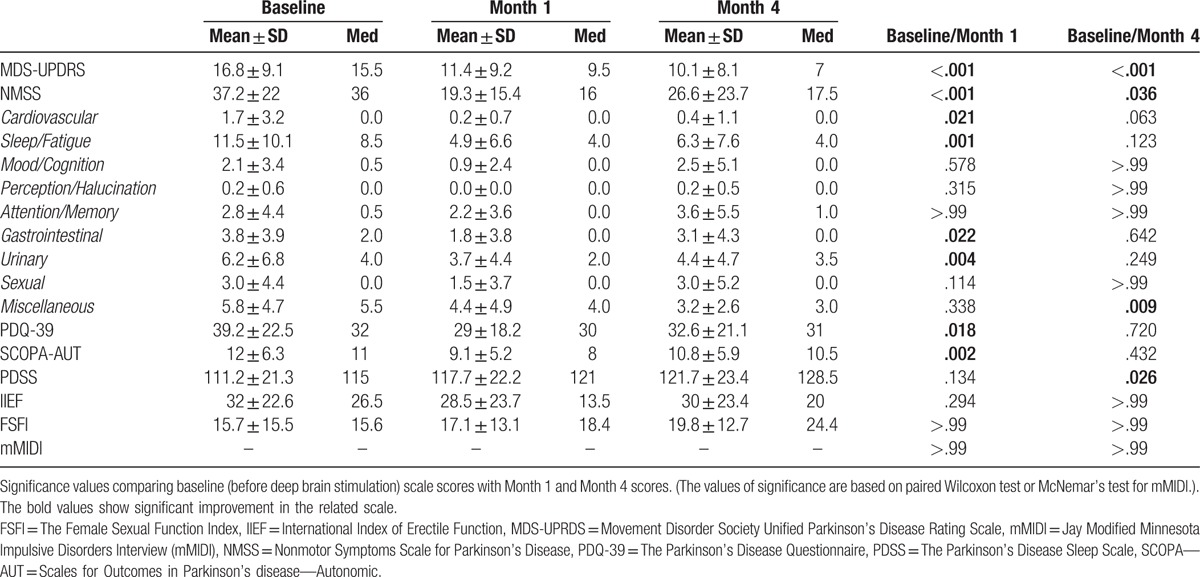
Differences in motor and nonmotor symptoms scores between baseline (preoperatively), Month 1, and Month 4 following DBS implantation.

Bilateral STN-DBS in patients with PD at M1 significantly reduced PDQ-39 scores (*P = *.018) and SCOPA-AUT scores (*P = *.002). But 4 months after implantation, the PDQ-39 and SCOPA-AUT scores were again increased, and the difference in the value before the stimulation was no longer statistically significant.

NMSS scores improved significantly at M1 (*P < *.001); at Month 4, the scores remained significantly lower than before stimulation (*P = *.036). The Cardiovascular, Sleep/Fatigue, Gastrointestinal, and Urinary subscores were significantly reduced at Month 1, but at Month 4 only the Miscellaneous score had a significant reduction (Table 2).

There was no significant difference in PDSS scores between baseline and Month 1 after DBS implantation, but there was a significant increase in PDSS score at Month 4 (*P = *.026).

MDS-UPDRS Part III scores show a significant improvement at Month 1 (*P < *.001) and at Month 4 after DBS implantation (*P < *.001).

DBS treatment resulted in no significant changes in FSFI or in IIEF at Month 1 or at Month 4.

Impulse control disorder (ICD) was present in only 4 patients, so we do not list the results as they cannot be considered relevant.

## 4 Discussion

- MDS-UPDRS, part III: Motor Examination
  The improvements, that is, the differences between the values at baseline and Months 1 and 4 in this scale were highly significant, as could be expected since STN-DBS was primarily developed and is indicated for the treatment of motor complications of Parkinson's disease.^[20]^
- Nonmotor Symptoms Scale for Parkinson's Disease
  A comparison of the overall NMSS values showed marked improvements at M1 and M4, with significance of < .001 and .036, respectively. When the individual domains were analysed, significant differences were present only in the following domains: Cardiovascular, Sleep/Fatigue, Gastrointestinal, and Urinary at M1; only in Miscellaneous at M4. It is hard to speculate whether the improvements in these domains were caused by the lesional effect of the STN implantation, or whether it is a behavioral rebound effect of the patient's positive expectations. We favor the lesional effect in the Sleep/Fatigue domain, because this has been reported in previous studies, although some of them were conducted on smaller cohorts.^[21]^ Nevertheless, a heuristic analysis for this NMSS domain should be conducted, since the results in our group were different when they were compared with the PDSS (PDSS improvement was not significant at M1 and highly significant at M4). Improvement in the functioning of visceral organs has rarely (if ever) been noted in connection with DBS treatment, so we suspect a dominant role of positive treatment expectation here, as this has been repeatedly reported for cognitive and even motor functions.^[22]^ Improvement in the Miscellaneous—olfactory function and excessive sweating subdomains—has been recently reported, and we support the authors’ explanation of this effect, although their patients were assessed 6 months following the start of stimulation.^[23]^
- The Parkinson's Disease Questionnaire
  An initial significant improvement in the PDQ-39 value in M1 was followed by a return to practically identical values as before surgery in M4. This fact is in our opinion a holistic reflection of the fact that quality of life is significantly affected by patients’ expectations. The initial postsurgical improvement, still present at the M1 visit, was caused by the positive expectations of patients who believed that the stimulation process would substantially improve their motor symptoms. The slow and only gradual course of improvement over the first 3 months of stimulation changed the original position in the patient/doctor/treatment pattern, and thus the PDQ-39 score at M4 may reflect incompletely fulfilled expectations.
- SCOPA-AUT Questionnaire
  Previous studies used different tools to assess autonomic functions,^[24,25]^ so it is difficult to compare their results from relatively small groups of patients with our results. Nevertheless, we recorded a highly significant improvement following the surgery (M1), and a subsequent return to the values before surgery at M4. This difference can be explained by either lesional effects of the implantation,^[26]^ or by common positive expectations, which may afflict the functions assessed in the SCOPA-AUT scale.^[27,28]^
- The Parkinson's Disease Sleep Scale
  Our PDSS results correlate with the results of the 8 studies reported in a recent meta-analysis.^[29]^ In 6 of these studies, mean PDSS score was significantly improved (16–41%) following DBS implantation; this improvement was present in all studies at 4 weeks after surgery, and remained stable for the next 6 months. It has been speculated that STN stimulation directly affects sleep physiology via anatomical connections of the STN with the pedunculopontine nucleus, nucleus raphe, and laterodorsal tegmental nucleus. Taking into account the incomplete evidence of the role of STN in these anatomical structures, we prefer the explanation that the sleep improvement was caused by the significant alleviation of night motor complications, namely off-states and painful early morning dystonia, similarly as in apomorphine treatment.^[30,31]^
- International Index of Erectile Function
  In our group, there was no significant difference or trend between the mean values of IIEF at baseline and Month 1 and Month 4. This finding contradicts previous studies as listed in the review by Tykocki et al.^[32]^ Several reports highlighted hyper-sexuality induced by STN-DBS, and one study^[33]^ found evidence of improved sexual functioning after STN-DBS. Why this effect was not found in our patient population (20 males) is not clear; the relatively short period of stimulation may be a substantial factor. As there are similarities between DBS and dopaminergic stimulation, it is worth mentioning our study with pergolide, in which the improvement in the IIEF scale was present only after 6 months of treatment.^[34]^
- The Female Sexual Function Index
  Previous reports did not note any significant improvement of sexual dysfunction in female subjects undergoing STN-DBS.^[33]^ The number of female subjects in our study was very low, so the results are not relevant.
- Jay Modified Minnesota Impulsive Disorders Interview
  Some studies have reported improved impulse control disorder after STN-DBS, as assessed by clinical diagnostic interview and neuropsychological examination.^[35]^ In our group, there were only 3 patients with recorded impulse control disorder (gambling, punding, and compulsive shopping). Despite the individual improvement following the start of DBS treatment, the number of patients is too low to comment on the improvement measured with this scale.

## 5 Limitations of the study

There are several limitations of our study to be considered. Firstly, we have to mention that some of the scales we used are just self-administered questionnaires which are trying to present a holistic assessment of nonmotor symptoms.

We also have to admit the option that some of the patients might be reluctant to reveal certain NMS in a clinical setting and so the results can be inaccurate.

Also, the impact of the reduction of oral medication on the appearance of nonmotor symptoms has not been fully elucidated yet. Nevertheless, practically none of the NMS, which has been afflicted in the STN-DBS treatment in our study, is caused by the dopaminergic medication, all of them are generally seen as a symptom of the disease itself.^[36]^

Using short-term follow-up investigations minimizes the impact that disease progression could have on NMS and enables us to address the research question in a relatively short time span. However, the optimal stimulating parameters and medical equilibration can be achieved 6 months after STN-DBS or even later, meaning that many of the patients are evaluated postoperatively in suboptimal stimulation conditions. In these cases, the impact of DBS on several NMS is likely to be vastly underestimated.^[29]^

## 6 Conclusions

Our pilot study provides evidence that deep brain stimulation of the subthalamic nucleus in patients with advanced, complicated Parkinson's disease quickly improves not only motor symptoms, but also several domains of nonmotor functions, namely sleep, autonomic functions and quality of life, and this improvement is present immediately following the start of stimulation. Whether other nonmotor domains remain intact will be assessed in a further study with a significantly higher number of subjects.

## References

[1] Faggiani E Benazzouz A Deep brain stimulation of the subthalamic nucleus in Parkinson's disease: from history to the interaction with the monoaminergic systems. Prog Neurobiol 2016;151:139–56. 27412110 10.1016/j.pneurobio.2016.07.003

[2] Timmermann L Jain R Chen L Multiple-source current steering in subthalamic nucleus deep brain stimulation for Parkinson's disease (the VANTAGE study): a non-randomised, prospective, multicentre, open-label study. Lancet Neurol 2015;14:693–701. 26027940 10.1016/S1474-4422(15)00087-3

[3] Sauerbier A Ray Chaudhuri K Non-motor symptoms: the core of multi-morbid Parkinson's disease. Br J Hosp Med 2014;75:18–24. 10.12968/hmed.2014.75.1.18 24401966

[4] Noyce AJ Lees AJ Schrag AE The prediagnostic phase of Parkinson's disease. J Neurol Neurosurg Psychiatry 2016;87:871–8. 26848171 10.1136/jnnp-2015-311890 PMC4975823

[5] Pfeiffer RF Non-motorsymptoms in Parkinson's disease. Parkinsonism Relat Disord 2016;22:119–22. 10.1016/j.parkreldis.2015.09.004 26372623

[6] Cury RG Galhardoni R Teixeira MJ Subthalamic deep brain stimulation modulates conscious perception of sensory function in Parkinson's disease. Pain 2016;157:2758–65. 27559833 10.1097/j.pain.0000000000000697

[7] Ineichen C Baumann-Vogel H Christen M Deep brain stimulation: in search of reliable instruments for assessing complex personality-related changes. Brain Sci 2016;6:E40. 27618110 10.3390/brainsci6030040 PMC5039469

[8] Nassery A Palmese CA Sarva H Psychiatric and cognitive effects of deep brain stimulation for parkinson's disease. Curr Neurol Neurosci Rep 2016;16:87. 27539167 10.1007/s11910-016-0690-1

[9] Wolz M Hauschild J Koy J Immediate effects of deep brain stimulation of the subthalamic nucleus on nonmotor symptoms in Parkinson's disease. Parkinsonism Relat Disord 2012;18:994–7. 22682974 10.1016/j.parkreldis.2012.05.011

[10] Goetz CG Tilley BC Shaftman SR Movement Disorder Society UPDRS Revision Task Force. Movement Disorder Society—sponsored revision of the Unified Parkinson's Disease Rating Scale (MDS-UPDRS): scale presentation and clinimetric testing results. Mov Disord 2008;23:2129–70. 19025984 10.1002/mds.22340

[11] Fahn S Elton RL Fahn S Marsden CD Goldstein M UPDRS program members. Unified Parkinson's Disease Rating Scale. Recent Developments in Parkinson's disease, vol. 2. Florham Park, NJ: Macmillan Healthcare Information; 1987 153–63.

[12] Chaudhuri KR Martinez-Martin P Brown RG The metric propertiesof a novel non-motor symptoms scale for Parkinson's disease: Results from an international pilot study. Mov Disord 2007;22:1901–11. 17674410 10.1002/mds.21596

[13] Peto V Jenkinson C Fitzpatrick R The development and validation of a short measure of functioning and well being for individuals with Parkinson's disease. Qual Life Res 1995;4:241–8. 7613534 10.1007/BF02260863

[14] Jenkinson C Fitzpatrick R Peto V The Parkinson's Disease Questionnaire (PDQ-39): development and validation of a Parkinson's disease summary index score. Age Ageing 1997;26:353–7. 9351479 10.1093/ageing/26.5.353

[15] Visser M Marinus J Stiggerlbout AM Assessment of autonomic dysfunctions in Parkinson's disease: the SCOPA-AUT. Mov Disord 2004;19:1306–12. 15390007 10.1002/mds.20153

[16] Chaudhuri KR Pal S DiMarco A Parkinson's disease sleep scale: a new instrument for assessing sleep and nocturnal disability in Parkinson's disease. J Neurol Neurosurg Psychiatry 2002;73:629–35. 12438461 10.1136/jnnp.73.6.629 PMC1757333

[17] Rosen RC Riley A Wagner G The international index of erectile function (IIEF): a multidimensional scale for assessment of erectile dysfunction. Urology 1997;49:822–30. 9187685 10.1016/s0090-4295(97)00238-0

[18] Rosen R Brown C Heiman J The Female Sexual Function Index (FSFI): a multidimensional self-report instrument for the assessment of female sexual function. J Sex Marital Ther 2000;26:191–208. 10782451 10.1080/009262300278597

[19] Christenson GA Faber RJ de Zwaan M Compulsive buying: descriptive characteristics and psychiatric comorbidity. J Clin Psychiatry 1994;55:5–11. 8294395

[20] Chou KL Taylor JL Patil PG The MDS-UPDRS tracks motor and non-motor improvement due to subthalamic nucleus deep brain stimulation in Parkinson's disease. Parkinonism Relat Disord 2016;19:966–9. 10.1016/j.parkreldis.2013.06.010 PMC3825788 23849499

[21] Merlino G Lettieri C Mondani M Microsubthalamotomy improves sleep in patients affected by advanced Parkinson's disease. Sleep Med 2014;15:637–41. 24784787 10.1016/j.sleep.2013.12.016

[22] Keitel A Wojtecki L Hirschmann J Motor and cognitive placebo-nocebo-responses in Parkinson's disease patients with deep brain stimulation. Behav Brain Res 2013;250:199–205. 23651878 10.1016/j.bbr.2013.04.051

[23] Dafsari HS Reddy P Herchenbach C IPMDS Non-Motor Symptoms Study Group. Beneficial effects of bilateral subthalamic stimulation on non-motor symptoms in Parkinson's disease. Brain Stimul 2016;9:78–85. 26385442 10.1016/j.brs.2015.08.005

[24] Ludwig J Remien P Guballa C Effects of subthalamic nucleus stimulation and levodopa on the autonomic nervous system in Parkinson's disease. J Neurol Neurosurg Psychiatry 2007;78:742–5. 17371906 10.1136/jnnp.2006.103739 PMC2117689

[25] Halim A Baumgartner L Binder DK Effect of deep brain stimulation on autonomic dysfunction in patients with Parkinson's disease. J Clin Neurosci 2011;18:804–6. 21489800 10.1016/j.jocn.2010.10.015

[26] Basiago A Binder DK Effects of deep brain stimulation on autonomic function. Brain Sci 2016;6:E33. 27537920 10.3390/brainsci6030033 PMC5039462

[27] Evatt ML Chaudhuri KR Chou KL Dysautonomia rating scales in Parkinson's disease: sialorrhea, dysphagia, and constipation-critique and recommendations by movement disorders task force on rating scales for Parkinson's disease. Mov Disord 2009;24:635–6. 19205066 10.1002/mds.22260 PMC4404514

[28] Rodriguez-Blazquez C Forjaz MJ Frades-Payo B Longitudinal Parkinson's Disease Patient Study, Estudio Longitudinal de Pacients con Enfermedad da Parkinson Group. Independent validation of the scales for outcomes in Parkinson's disease-autonomic (SCOPA-AUT). Neur J Neurol 2010;17:194–201. 10.1111/j.1468-1331.2009.02788.x 19780808

[29] Eugster L Bargiotas P Bassetti CL Deep brain stimulation and sleep-wake functions in Parkinson's disease: a systematic review. Parkinsonism Relat Disord 2016;32:12–9. 27605426 10.1016/j.parkreldis.2016.08.006

[30] Kanovský P Kubová D Bares M Levodopa-induced dyskinesias and continuous subcutaneous infusions of apomorphine: results of a two-year, prospective follow-up. Mov Disord 2002;17:188–91. 10.1002/mds.1276 11835461

[31] Garcia Ruiz PJ Nocturnal subcutaneous apomorphine infusion for severe insomnia in Parkinson's disease. Mov Disord 2006;21:727–8. 16547919 10.1002/mds.20852

[32] Tykocki T Mandat T Nauman P Influence of subthalamic deep brain stimulation on dysautonomia observed in Parkinson's disease. Neurol Neurochir Pol 2010;44:277–84. 20625964 10.1016/s0028-3843(14)60042-6

[33] Castelli L Perozzo P Genesia ML Sexual well being in parkinsonian patients after deep brain stimulation of the subthalamic nucleus. J Neurol Neurosurg Psychiatry 2004;75:1260–4. 15314111 10.1136/jnnp.2003.034579 PMC1739238

[34] Pohanka M Kanovský P Bares M Pergolidemesylate can improve sexual dysfunction in patients with Parkinson's disease: the results of an open, prospective, 6-month follow-up. Eur J Neurol 2004;11:483–8. 15257688 10.1111/j.1468-1331.2004.00820.x

[35] Merola A Romagnolo A Rizzi L Impulse control behaviors and subthalamic deep brain stimulation in Parkinson disease. J Neurol 2017;264:40–8. 27761641 10.1007/s00415-016-8314-x

[36] Dafsari HS Reker P Stalinski L EUROPAR and the IPMDS (International Parkinson's and Movement Disorders Society) Non-Motor Parkinson's Disease Study Group. Quality of life outcomes after subthalamic stimulation in Parkinson's disease depends on age. Mov Disord 2017;33:99–107. 29150860 10.1002/mds.27222

